# A Reappraisal of the Evolutionary and Developmental Pathway of Apomixis and Its Genetic Control in Angiosperms

**DOI:** 10.3390/genes11080859

**Published:** 2020-07-28

**Authors:** Gianni Barcaccia, Fabio Palumbo, Sergio Sgorbati, Emidio Albertini, Fulvio Pupilli

**Affiliations:** 1 Department of Agronomy Food Natural Resources Animals Environment, University of Padova, Campus of Agripolis, Viale dell’Università 16, Legnaro, 35020 Padova, Italy; fabio.palumbo@unipd.it; 2 Department of Environmental and Territory Sciences, University of Milano-Bicocca, Piazza della Scienza 1, 20126 Milano, Italy; sergio.sgorbati@unimib.it; 3 Department of Agricultural, Food and Environmental Sciences, University of Perugia, 06121 Perugia, Italy; emidio.albertini@unipg.it; 4 Research Division of Perugia, Institute of Biosciences and Bioresources, National Research Council (CNR), Via Madonna Alta 130, 06128 Perugia, Italy; fulvio.pupilli@ibbr.cnr.it

**Keywords:** agamospermy, basal angiosperms (ANA-grade), sporocyteless, polycomb-group proteins, reproductive systems, apomixis evolution

## Abstract

Apomixis *sensu stricto* (agamospermy) is asexual reproduction by seed. In angiosperms it represents an easy byway of life cycle renewal through gamete-like cells that give rise to maternal embryos without ploidy reduction (meiosis) and ploidy restitution (syngamy). The origin of apomixis still represents an unsolved problem, as it may be either evolved from sex or the other way around. This review deals with a reappraisal of the origin of apomixis in order to deepen knowledge on such asexual mode of reproduction which seems mainly lacking in the most basal angiosperm orders (i.e., Amborellales, Nymphaeales and Austrobaileyales, also known as ANA-grade), while it clearly occurs in different forms and variants in many unrelated families of monocots and eudicots. Overall findings strengthen the hypothesis that apomixis as a whole may have evolved multiple times in angiosperm evolution following different developmental pathways deviating to different extents from sexuality. Recent developments on the genetic control of apomixis in model species are also presented and adequately discussed in order to shed additional light on the antagonist theories of gain- and loss-of-function over sexuality.

## 1. Introduction and Background Information

Apomixis *sensu stricto* (agamospermy) is asexual reproduction by seed [1]. For eukaryotes in general, apomixis is life cycle renewal through gamete-like cells that give rise to maternal embryos but without sexuality and sex, that is, without ploidy reduction (i.e., meiosis) and ploidy restitution (i.e., syngamy). Both meiosis and syngamy are canalisations of complex molecular processes that have remained conserved among single-celled and, later, multi-celled species of eukaryotes since eukaryogenesis [2,3,4,5,6,7,8]. Apomixis, like sex, is kingdom ubiquitous occurring in thousands of species across eukaryotes [9,10,11].

Since apomictic reproduction involves the development of an embryo from an apomeiotic or somatic cell with a maternal genome, there are several ways to produce seeds of apomictic origin. This process can have a nucellar or integumental initiation, depending on the tissue of the ovule from which the maternal cell with embryonic competence differentiates.

The simplest pathway, known as adventitious embryony, avoids the production of a gametophyte and one or more vegetative embryos form within the nucellus or the integument. This phenomenon seems to have evolved more frequently in tropical than in temperate flora [12,13] and to be more represented in diploid species, while other forms of apomixis are more frequent in polyploids. Adventitious embryony is found in several non-agriculturally important species, with the exception of several *Citrus* species and mango (*Mangifera indica*) [14].

Another pathway, known as gametophytic apomixis, occurs when the maternal embryo originates from an apomeiotic egg cell differentiated into an unreduced embryo sac [15] arisen from a somatic nucellar cell that acquires the developmental program of a functional megaspore (apospory) or from a megaspore mother cell with suppressed or modified meiosis (diplospory). It is worth nothing that the gametic cell fate in apomictic plants can be activated either in somatic cells (apospory) or in unreduced megaspores (diplospory) as surrogate for meiotic products [6,16].

Sexual reproduction is based on the alternation of a diploid (sporophytic) and haploid (gametophytic) generation, both of which are fringed by events that lead to a change in ploidy, i.e., meiosis and fertilization. In gametophytic apomixis, both edge events are short-circuited as the egg cells originate through mitosis (apospory) or restitutional meiosis (diplospory), preserve a maternal genomic composition, and the embryos develop autonomously without any contribution of a spermatic nucleus (parthenogenesis). This combination was referred to as recurrent apomixis [15] as the original genotype may be indefinitely reiterated over generations.

Scaling up and down the ploidy level by means of genome accumulation or limitation can eventually take place by unreduced egg cell fertilization or reduced egg cell parthenogenesis respectively. These reproductive strategies have been referred to as non-recurrent apomixis [15]. Although not offering a stable means for genotype propagation, apomictic variants have likely been an important player in the evolution of polyploid species.

## 2. The Evolution of Apomixis

From the evolutionary point of view, the nature and persistence of asexual reproduction remains one of the most challenging phenomena [17]. In plants, asexuality (apomixis) either resurfaces in sexual lineages or it is derived by mutations from sexuality (amphimixis), and not only has it originated independently multiple times between different species but it has also resurfaced or evolved recurrently within certain species. Hence, genomic shocks, such as hybridization, polyploidization or both, have repeatedly switched from sexual to asexual reproduction. Despite the hypothesized disadvantages associated with apomixis, including limited genetic variation and mutation accumulation, asexually reproducing plants are highly adaptable, surprisingly stress tolerant and stable from an evolutionary perspective, and therefore questions regarding the origin and evolution of asexuality are still a matter of debate among population geneticists.

Species characterized by gametophytic apomixis are mainly polyploid, whereas their sexual relatives are usually diploid [18]. It is well known that many wild apomictic species are characterized not only by polyploidy, but also by hybridity [19]. In fact, apomicts usually belong to complex taxa whose members regularly undergo intra- and interspecific hybridization patterns. However, it is not yet clear what the relative contributions of hybridization and polyploidization models are, due to origin and evolution of the asexual lineage, since both phenomena can have different regulatory consequences [20,21,22], which could presumably lead to coordinated deregulation of stress perception and adaptation genes as well as the sexual path in a sexual ancestor. Moreover, an asexual pathway is obligate infrequently: most apomictic species are facultative, as individuals can set seeds through both sexual and apomictic reproductive modes.

Apomixis may now be regarded as a consequence of sexual failure (i.e., loss-of-function) rather than as a recipe for clonal success (i.e., gain-of-function). There is increasing evidence that apomixis is a modification of the normal sexual developmental pathway (reviewed by [1,23]). Most of the events that characterize sexual reproduction may be retained both structurally and functionally in apomictic reproduction, with the exceptions that the reduced egg cell is replaced by an unreduced egg cell, with absent or modified meiosis (i.e., apomeiosis), and the seed development does occur without egg cell fertilization (i.e., parthenogenesis). In addition, it is clear that residual sexual function is retained in pseudogamous apomixis; in fact, if it is true that seed development may occur without fertilization of either the egg cell or the central cell (i.e., autonomous apomixis), it is also true that fertilization may be required to form the endosperm in many apomictic plants [15].

Comprehension of parallel or convergent evolution of apomixis may be crucial for seeking the causal gene/s, as they represent distinctive labels for its independent resurfacing or origin among populations or species. The rationale usually suggests that, given a plant phenotype, the underlying genetic factors and mechanisms could be considered similar in closely related species or different in distantly related species. With particular reference to the mode of reproduction, i.e., apomixis vs. sexuality, the same reproductive strategy might have theoretically resurfaced or evolved among populations even within species by changes in different gene/s or among distantly related species by changes in the same gene/s through convergent or parallel evolution. As a general perspective, nowadays in the post-genomic era, reconsidering and understanding what we have learned about the genetic control of apomixis should help us to critically determine the evolution of this trait, including both apospory and diplospory, at least in the most studied model species.

If parallel evolution has occurred for apomixis, this reproductive strategy is expected to have evolved independently in different populations even in closely related species. In fact, different populations or species showing similar patterns of development are likely modified in similar ways if subjected to similar selection pressures. Hence, “parallelism” refers to independent developmental modifications of the same kind that gives rise to similar phenotypes. As a matter of fact, because closely related species have similar developmental programs, parallelism is frequent among phylogenetically related organisms [24]. With convergent evolution, different developmental pathways may generate similar phenotypes. This means that “convergence” includes independently evolved features that are similar as manifested trait, but have arisen by different developmental pathways [24]. If so, apomixis is expected to have arisen from different genetic modifications and developmental programs that have then evolved similarly even in distantly related organisms.

Apomixis has been detected in ferns (where this is better referred as to apogamy, i.e., development of a sporophyte from a gametophyte without fertilization), is rare in gymnosperms and very common in angiosperms [25]. Apomictic fern lineages have been documented only in recent years [26], distinguishing between two pathways to diploid spore production (premeiotic endomitosis, more common, and meiotic first division restitution, less frequently observed). Each of these two alternative spore-generating pathways yields chromosomally unreduced spores (i.e., diplospores) which then germinate. Within leptosporangiate ferns, nearly half of the families contain one or more apomictic taxa [26]. Interestingly, the frequency of apomixis seems significantly correlated with species diversity, but any significant relationship was found between apomixis and diversification rates [27]. These findings are in agreement with studies showing that apomictic lineages are generally youthful, with initial estimates placing the ages of extant apomictic ferns in relatively recent evolutionary time, most having appeared within 8 Ma [27] or 15 Ma [28].

**Figure 1:**
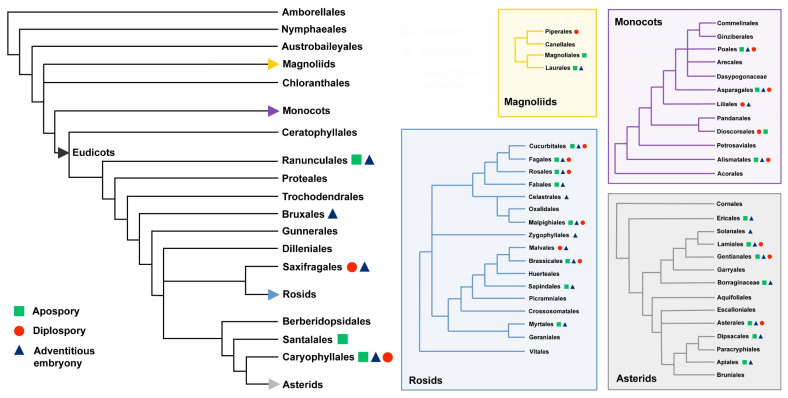
Distribution of the main types of apomixis (i.e., apospory, diplospory and adventitious embryony) among the main clades, orders and families of flowering plants. Detailed information for subclusters belonging to Magnoliids, Monocots, Rosids and Asterids are reported in the coloured boxes. Experimental evidences on apomictic pathways for taxonomic units are derived from [10,29] while phylogenetic trees are modified from [30].

In flowering plants, apomixis has been detected and documented in at least 79 families and 292 distinct genera [10] (Figure 1).

Moreover, there are certain angiosperm families that show a great deal of apomixis affecting several genera: the outstanding examples are in the Rosaceae, Poaceae (Graminae) and Asteraceae (Compositae). Because, in these families, gametophytic apomixis, by means of either apospory or diplospory, is phylogenetically clustered above the genus level, it is argued that some clades, including closely related species with common ancestry within eudicots and monocots, may be preadapted and inclined to let apomixis evolve more easily [25]. With particular reference to Rosaceae, the phylogenetic origin and taxonomic distribution of apomixis have recently being investigated in great details in tribe Potentilleae [31]. Regular sexuality was distinguished from apomixis, as well as the zygotic versus parthenogenic origin of embryos and the pseudogamous versus autonomous origin of the endosperm. Records on the reproductive mode were provided for the genus *Farinopsis*, including 29 species belonging to five genera and seven series of *Potentilla*. Regular sexuality was observed in *Aphanes*, *Argentina*, *Comarum*, *Dasiphora*, *Drymocallis*, *Farinopsis*, *Fragaria*, *Horkeliella*, *Potentilla* and *Sibbaldia*, whereas apomixis was restricted to two evolutionary lineages: the *Potentilla* core group and *Alchemilla*/*Aphanes*. Early evolutionary divergence of these lineages (approximately 50 Ma), characterized by pseudogamous and autonomous apomictic seed formation, respectively, suggested parallel origins of apomixis [31]. Such a parallel origin would be the consequence of a repeated evolution of the same phenotype or genotype in different populations. Moreover, apomixis is shown to be taxonomically widespread in the whole Northern Hemisphere distribution range of *Potentilla*, a pattern that is explained by interspecific hybridization/introgression events and repeated intercontinental dispersals.

Furthermore, apomicts and their sexual relatives are often sympatric and thus frequently have overlapping ranges of adaptation. More specifically, compared to their sexual relatives, apomictic plants typically have wider geographical ranges, which extend into higher latitudes and altitudes, and better abilities to re-colonize regions after glaciation [32]. Despite a number of factors, i.e., the Pleistocene origins of apomixis—together with hybridity and polyploidy, unidirectional gene flow, niche targeting by asexual clones and limited biotic interactions in regions of glaciations—probably may be accounted for the geographical differences between sexual and apomictic plants; the relative influence of each is probably species-specific [33].

In the case the two main different variants of apomixis, i.e., apospory and diplospory, these do not have a common ancestor for the two major angiosperm lineages, i.e., monocots and eudicots; as it seems most likely because of the very differentiated developmental routes and taxonomic entities, then apomixis has arisen independently many times in the evolution of angiosperms. In addition, considering the two most widespread routes to originate the unreduced gametophyte, it is also apparent that the diplosporous condition involves a less radical departure from the normal sexual pattern that does the aposporous one (reviewed by [23]).

In the last 20 years, fossil records and morphological and molecular analyses involving extant taxa have clarified the position of many clades inside the angiosperms phylogenetic tree [34]. Three clades, Amborellaceae, Nymphaeales and Austrobaileyales (ANA-grade), are now considered “basal angiosperms” or “basalmost angiosperms”, sisters to all other angiosperms or “mesangiosperms”, representing an important information to infer ancestral morphological, chemical and genomic attributes to the earliest angiosperms [35,36,37,38,39]. The extant basal angiosperms may not be representative of the earliest, now extinct lineages. However, the large diversification (woody and terrestrial, herbaceous and aquatic, annual and perennial, lacking vessels or not, with different types of leaves, phyllotaxis, flowers and reproductive biology) of the relative few extant and basalmost angiosperms (about 200 species), in addition to fossil records, could be representative of a much larger diversity of early and now extinct lineages [34]. Particularly interesting is the presence, inside the ANA grade, of three different patterns of female gametophyte development: *Amborella*–type, *Nuphar-Schizandra* type and *Polygonum* type [40]. A large variety of breeding systems is also present: *Amborella trichopoda* is dioecious, *Trithuria* (Hydatellaceae, 12 species) possesses a mixture of dioecious, monoecious and bisexual breeding systems, whereas the remaining Nymphaeales (91 species) show bisexual flowers. In addition, within Austrobaileyales, *Austrobaileya scandens* is bisexual, *Trimenia* (8 species) contains both andromonoecious and bisexual species, whereas in the Schizandraceae family *Illicium* (44 species) is fully bisexual, *Schisandra* (25 species) contains a mixture of monoecious and dioecious species and *Kadsura* (16 species) is predominantly monoecious, with a tendency toward dioecious behaviours [41]. In general, outcrossing systems are prevalent, some species have common adaptation to promote selfing, whereas only two species (*Trithuria filamentosa* and *T. incospicua*, Hydatellaceae) may putatively hide apomictic features [42,43], even if several other modes of asexual propagation are exhibited, including vegetative proliferation by tubers, stolons and other foliar parts [44,45].

Polyploidy by whole genome duplication (WGD), followed by gene loss and diploidization is spread along almost all fundamental lineages of the angiosperm phylogenetic tree and is generally considered to be a common mode of speciation that has far-reaching consequences for plant macroevolution and ecology [46,47]. Intragenomic syntenic analysis within Spermatophyta provides clear structural evidence of an ancient genomic duplication event shared by all flowering plants in a (difficult to estimate) time in 300–200 Mya, leading to the most recent common ancestor of extant angiosperms [48,49]. Establishment of a newly arisen apomictic lineage is often fostered by side-effects of polyploidy [50]. As for basal angiosperms (ANA grade), no evidence of WGD and apomictic mechanisms were found in the *Amborella* species and Austrobayleales lineages, whereas a WGD event was reported in water lilies (Nymphaeales) lineage [51] that would reveal the putative presence of apomictic species in *Trithuria* genus [43].

In conclusion, based on available experimental evidences, the three main types of agamospermy occur in all major clades of flowering plants, adventitious embryony being the most frequent form (148 genera), followed by aposporous apomixis (110 genera) and diplosporous apomixis (68 genera) [29]. Since the vast majority of historical records only reported the most prevalent type, we cannot rule out the coexistence of at least two if not all three main types of apomictic forms, because this is known as a common feature in apomictic plants [52]. In addition, with specific reference to the most basal angiosperm, *Amborella trichopoda*, apomixis is not occurring in this species and it was never documented in other non-flowering seed plant lineages, including gymnosperm species [29]. The only exception is represented by *C. dupreziana* (Pinophyta, Cupressaceae), where a particular case of paternal apomixis is thought to have evolved from sexuality in response to the reduction of population size, apparently limited to a few hundreds of individuals [53]. However, this type apomixis, where the embryo seems to result from the development of diploid pollen, is hardly comparable to the forms of apomixis observed within the Angiosperms clade.

## 3. The Genetic Control of Apomixis: A General Overview

One of the major challenges of population biology is to understand which are the genetic determinants that control the maintenance of sexual reproduction under natural selection. In this view, the fundamental components of amphimixis, such as genetic recombination and gamete fusion, allow the single individuals of a population to experience new allelic combinations and interactions leading to diversification and adaptation. Nevertheless, alternative routes of reproduction that circumvent sex, such as apomixis, gained significant evolutionary success [54]. In the offspring of apomictic plants, genetic diversity is avoided or minimized, as the embryos retain the maternal genotype and their development is independent from both meiotic reduction and egg cell fertilization in ovules. Fertilization of the central cell is often required for endosperm formation (pseudogamy).

During the last two decades, many scientists have worked on the isolation of the genetic determinants of the apomictic pathway with the perspective to induce apomictic reproduction in crop plants by genetic engineering (for review, see [16,18]). More recently, although artificially induced apomictic rice has been obtained [55] using a synthetic approach (e.g., by engineering key regulator genes of sexual development), additional research is required to determine stability of induced apomictic reproduction in field conditions. Consequently, even now using the modern tools of genomics, understanding the molecular pathway leading to apomixis in natural apomicts is necessary, but more complicated than expected.

Nowadays, new insights have contributed to shed light into the structural and functional feature of apomixis; these include the structural parallelism between the apomixis controlling region (ACR) in several natural apomicts and the Y-chromosome of dioecious plants [18], the silencing mechanism of a specific apomixis-linked genetic factor [56] and the functional validation of a genetic determinant of parthenogenesis in *Pennisetum squamulatum* [57].

Recent advances, based on sequencing, in silico mapping and in vitro expression analysis of selected apomixis-linked genes allowed the identification of a chromosome area common to *Sorghum bicolor*, *Setaria italica*, *Brachypodium distachyum*, rice and maize syntenic to the apomixis locus of *Paspalum simplex* [58]. This synteny group revealed different extents of gene collinearity with the apomixis locus, including genes with well-defined annotations for biological processes and molecular functions. Most importantly, apomixis-linked genes were expressed as both sense and antisense mRNAs and both transcripts proved to be more abundant in sexual compared to apomictic ovules, indicating a putative silencing effect of the apomixis-linked alleles on their sexual-specific counterparts in these cells [58]. This finding could act in favour of apomixis surfacing or evolved by silencing sex genes.

More specifically, it would appear that there are at least two distinct elements in the control of gametophytic apomixis: the production of unreduced embryo sacs, originating through apospory or diplospory, and the subsequent development of the embryos through parthenogenesis. However, other elements may need to be incorporated into a model explaining/miming the genetic control of apomixis, including pseudogamy and, in some apomicts, the autonomous development of endosperm.

Apomeiosis is rather a rare phenomenon when uncoupled with parthenogenesis while it is frequently observed as formation of unreduced gametes to overcome sterility of inter-specific hybrids leading to the origin and evolution of polyploid forms [59,60,61]. Conversely, when apomeiosis is coupled with parthenogenesis it attains regular elevated expression in natural apomicts [62]. Furthermore available data suggest that in some cases apomeiosis, by either apospory or diplospory, may be functionally and genetically independent from parthenogenesis and autonomous endosperm formation [16,18]. Finally, in most apomicts, both apospory and diplospory proved to be simply inherited in populations segregating for apomixis and a complex genetic model based on delicate interactions between initiators and repressors of both apomeiosis and parthenogenesis has been proposed for several species [63,64].

Analysis of genetic and molecular studies of apomicts is provided by several reviews, including those of Ozias-Akins and Conner [65], Hojsgaard [66], Whitton et al. [12], Barcaccia and Pupilli [18] Hand and Koltunow [1] and Schmidt [67]. Both naturally occurring and induced mutants holding individual components of apomixis have been identified (e.g., [68,69]), meaning that many taxa can potentially express apomixis-related traits, and that each component is under independent control and regulation. In several natural apomicts, the existence of genotypes which express only one component of apomixis or suppress the other (reviewed by [16]), further supports the hypothesis that distinct genetic factors control apospory, diplospory and parthenogenesis. It has long been recognized that apomixis is under control of single-dose dominant alleles (if hypothetically, apomixis were to be controlled by recessive alleles, then multiple-copies would be necessary in polyploids). Indeed, apomixis is inherited as a dominant trait in several apomictic species (see [70], for a review). However, even in species with simple inheritance patterns it is doubtful whether a single gene controls apomixis. As a matter of fact, the occurrence of genotypes that form embryos either from fertilized apomeiotic egg cells or by parthenogenic development of meiotically reduced egg cells have been documented by cyto-histological and flow-cytometric analyses, suggesting that apomeiosis and parthenogenesis may be uncoupled [16]. Recombinants for apomixis components that lack either apomeiosis or parthenogenesis have been reported in *Taraxacum officinale* [71], *Erigeron annuus* [72], *Poa pratensis* [73,74], *Hypericum perforatum* [75], *Ranunculus* [76] and *Cenchrus* species [77]. Finally, autonomous endosperm formation segregates independently from the other components of apomixis in *Hieracium*, [78,79]. On the whole, these findings suggest that apomixis may be controlled by a complex of closely linked genes.

A theoretical scenario for the origin and evolution of a two-gene apomixis system was proposed by Van Dijk and Vijverberg [45], including two dominant mutations that occur in a population of outcrossing hermaphrodites. A plant is changed by a first mutation from meiotic into apomeiotic, and by a second mutation from zygotic to parthenogenic embryo development. The chance that these two mutations would occur soon after each other within a nascent apomictic population seems unlikely [12]. There is, however, another possibility. Since most apomicts are of hybrid polyploid origin, perhaps the two mutations needed for the functioning of apomixis may be separately present in polyploid parent stocks and brought together by hybridization, leading to functional apomicts [23]. For the rise of apomixis in natural populations, an additional hypothesis calls the stepwise evolution model into question, as proposed by Hojsgaard and Hörandl [50]. It could be shown that sexual diploids have latent alleles for parthenogenesis with little or no penetrance, which become important benefiting from high expressivity if apomictic behaviour was introduced by specific mutations, giving rise to the development of apomeiotic egg cells. Moreover, for successful hybridization, a strict parthenogenic plant cannot function as a seed parent, since these mutations can only be combined in crosses between an apomeiotic seed parent and a parthenogenic male parent. This automatically results in polyploid apomictic hybrids, suggesting a direct relationship between gametophytic apomixis and polyploidy. New apomictic plants can function as a pollen donor in crosses with sexual plants, thereby generating new, secondary apomictic clones. This way would help explaining high clonal and genetic diversities, commonly found in populations of apomicts [80]. However, the main problem remains with this evolutionary scenario that the mutations for apomeiosis and parthenogenesis are individually deleterious and so they are expected to be selected against [81].

As reported by Briggs and Walters [23], devising a universal model of apomixis may be unrealistic: clearly this asexual mode of reproduction occurs in different forms and variants, and in many unrelated families of monocots and eudicots, suggesting that apomixis as a whole has evolved multiple times in angiosperm plant evolution following different developmental pathways, which perhaps are controlled by distinct genetic factors.

## 4. The Comparative Genomics of Master Genes

Nowadays, much is known about the formation of germlines in sexual plants, including genes specifying the cyto-genetic competence of sporocytes and driving the post-embryonic development. For instance, the earliest gene controlling this process in *Arabidopsis* is *SPOROCYTELESS* (*SPL*)/*NOZZLE* (*NZZ*). It encodes a transcriptional regulator of sporocyte development: loss of *SPL*/*NZZ* function changes the cell fate and abolishes the commitment to and initiation of sporogenesis in both male and female organs [82,83]. Since the gametophytic phase of the life cycle of all vascular plants (from ferns to angiosperms) starts with the development of haploid spores, this gene—known as essential for both male and female meiosis during sex organ development—is a master regulatory element of sexual plant reproduction.

Unfortunately, a similar gene that controls the primary step of apomixis is unknown and little is known about the mechanism that switches the reproductive process from sexual (i.e., meiotic) to asexual (i.e., apomeiotic). Many genes responsible for the formation of egg cells and unreduced embryo sacs, or involved in the development of the endosperm or embryo have been described, but a common apomictic pathway applicable to all crop plants has not been identified yet [16,18]. Hence, after two decades of substantial studies conducted in several laboratories and model plants, apomixis still seems to be an unsolved puzzle.

We know, for example, that *POLYCOMB GROUP* (*PcG*) genes, whose proteins exhibit some structural and functional conservation among higher plants, mammals and insects [84,85], are crucial for the development of multicellular organisms. Several variants well studied belong to the MEDEA-FERTILIZATION-INDEPENDENT ENDOSPERM (MEA–FIE) complex [86] that regulates cell proliferation during reproductive development, as meiotic products in flowering plants do not directly differentiate into gametes, but rather, they form the gametophytes, multicellular structures producing the gametes.

The endosperm and embryo developmental processes are both controlled by the female gametophyte at two different stages: repression of embryo/endosperm development in the absence of fertilization through imprinting, and expression of factors that are necessary after fertilization. In the absence of fertilization, the Arabidopsis FIE/FIS2/MEA complex—constituted by the FIS class PcG proteins MEDEA (MEA), FERTILIZATION-INDEPENDENT ENDOSPERM (FIE) and FERTILIZATION-INDEPENDENT SEED2 (FIS2)—suppresses endosperm development by regulating negatively the transcription of the genes directly involved in this process. Moreover, it has been shown that all mutations of the *fis* class of genes caused aberrant embryo and endosperm development if fertilized and exhibit autonomous endosperm development if unfertilized (reviewed by [18]). In any case, autonomously developed embryos and endosperms abort irrespectively from the paternal contribution. The proteins encoded by these genes mediate chromatin remodelling during seed formation. Imprinting, or parent-specific expression of genes, is a mechanism by which early stages of seed development are controlled by the female gametophytes.

**Figure 2:**
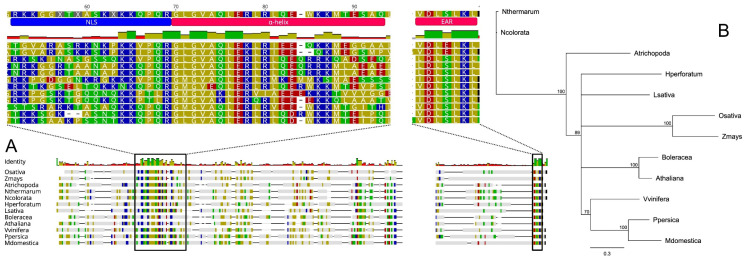
Bioinformatic analysis of the *SPL*/*NZZ* gene homologs in plant species representing the two major groups of angiosperms, monocotyledons and dicotyledons, including different basal angiosperm lineages belonging to the families Amborellaceae and Nymphaeaceae. (**A**) MUSCLE-based amino acid alignment of 11 putative SPL/NOZZLE sequences retrieved from *Zea mays* (KY110964.1), *Oryza sativa* (LOC_Os01g11430.1), *Hypericum perforatum* (apomictic species, OBUPD-D1 Hpctg51499), *Brassica oleracea* (Bol013057), *Malus domestica* (MD11G1234600), *Prunus persica* (Prupe.4G192500.1), *Vitis vinifera* (VIT_219s0014g03940.1), *Lactuca sativa* (Lsat_1_v5_gn_0_5400), *Amborella trichopoda* (XP_006833114.1), *Nymphaea colorata* (XP031473161.1), *Nymphaea thermarum* (KAF3782288.1), putative orthologues of SPL protein of *Arabidopsis thaliana* (AT4G27330). On the top of the panel the three main functional domains that resulted conserved among the 12 proteins are highlighted. NLS is a basic region rich in Arginine (R) and Lysine (K) that is thought to represent a putative nuclear localization signal; the α-helix sequence (also known as SPL-motif) is crucial for the constitution of homodimers and heterodimers, binding and inhibiting CINCINNATA (CIN)-like TEOSINTE BRANCHED1/CYCLOIDEA/PCF (TCP) transcription factors (TF); the EAR motif in the C-terminal region recruits TOPLESS/TOPLESS-RELATED (TPL/TPR) proteins to co-suppress the activity of the CIN-like TCP family. (**B**) Similarity-based neighbor-joining analysis performed using the 12 amino acid sequences with bootstrap values supporting all major nodes.

Here we investigated the *SPL*/*NZZ* gene homologs in plant species representing the two major groups of angiosperms, monocotyledons and dicotyledons, including different basal angiosperm lineages belonging to the families *Amborellaceae* and *Nymphaeaceae*. Based on their putative orthology (BLASTp; [87]) with the well-characterized SPL protein belonging to *Arabidopsis thaliana* (AT4G27330), we selected 11 amino acid sequences from *Zea mays* (KY110964.1)*, Oryza sativa* (LOC_Os01g11430.1), *Hypericum perforatum* (apomictic species, OBUPD-D1 Hpctg51499), *Brassica oleracea* (Bol013057)*, Malus domestica* (MD11G1234600), *Prunus persica* (Prupe.4G192500.1), *Vitis vinifera* (VIT_219s0014g03940.1), *Lactuca sativa* (Lsat_1_v5_gn_0_5400.1), *Amborella trichopoda* (XP_006833114.1), *Nymphaea colorata* (XP031473161.1) and *Nymphaea thermarum* (KAF3782288.1). Protein sequences were aligned (Geneious software v7.1.5, Biomatters, Ltd., Auckland, New Zealand) using MUSCLE [88] to highlight any conserved structures (Figure 2A). A similarity-based neighbour-joining analysis was also performed (Figure 2B).

Overall, sequences length ranged from 288 (*L. sativa*) to 386 amino acids (*Z. mays*) while the identity percentages resulting from all possible pairwise comparisons among proteins, varied from 10.7% (between classes, e.g., *Z. mays* vs. *B. oleracea*) to 97.8% (within genera, e.g., *N. thermarum* vs *N. colorata,* see Figure S1). Despite that most of the SPL-like sequences displayed high levels of inter-variability, the three main functional regions resulted in being conserved between dicots and monocots species. In the N-terminal region, as originally observed in *Arabidopsis* by Yang et al. [82] and Schiefthaler et al. [89], we detected a conserved basic region rich in Arginine (R) and Lysine (K) that is thought to represent a putative nuclear localization signal (NLS, [89]). A short α-helix sequence (also known as SPL-motif) immediately downstream of NLS and crucial for the constitution of homodimers and heterodimers both in vitro and in vitro [83] resulted also conserved among the sequences analysed. This region seems to be involved in binding and inhibiting CINCINNATA (CIN)-like TEOSINTE BRANCHED1/CYCLOIDEA/PCF (TCP) transcription factors (TF) whose activities are pivotal for both leaf development [90,91] and normal ovule development [92].

Finally, in the C-terminal region, a DLxLKL consensus sequence, previously described as ethylene-responsive element binding factor-associated amphiphilic repression (EAR) motif [83], characterized all the protein sequences here taken into consideration. Wei et al. [92] and Chen at el. [83] hypothesized that EAR motif is crucial to recruit TOPLESS/TOPLESS-RELATED (TPL/TPR) proteins and together they co-suppress the activity of the CIN-like TCP family, control the expression of other *TCP* genes and stimulate megasporocytes differentiation during ovule development.

Overall, the high conservation degree found in the three functional domains of the 12 SPL-like proteins analysed suggests a common role of this master gene in promoting megasporocytes differentiation both in monocotyledons and dicotyledons. Moreover, the identification of *SPL-like* sequences in *A. trichopoda*, *N. colorata* and *N. thermarum* and the lack of evidences of apomictic reproduction in these basalmost angiosperms species [29,93], support the hypothesis that apomixis may have evolved from sex. In the event that apomixis may not have stemmed from sexuality, the total absence of the *SPL/NZZ* gene was expected in apomictic species, as found for the *Hypericum perforatum* genome used as model, since the role of this gene in the initiation of the sexual pathway is now well consolidated.

Although originally thought to represent an ancient evolutionary predisposition manifested by specific taxa [19], a detailed phylogenetic analysis coupled with apomixis distribution recently performed by León-Martínez and Vielle-Calzada [29] suggests a multiple independent emergence and rapid spreading of apomixis among large families.

## 5. The Genetic Control of Apomixis

Apomictic reproduction has been detected in 78 out of 460 families of the flowering plants mainly in Rosaceae, Asteraceae and Poaceae and analysis of apomixis in current phylogenetic trees lead to the hypothesis that this trait evolved independently multiple times among plant families. In particular, in grasses for which genome collinearity has been largely demonstrated and apomixis is quite common, it has been hypothesized that this reproductive trait might be under the control of the same genes. It has been reported that apomixis controlling regions in grasses share intrinsic characteristics such as block of recombination and presence of high rate of mutations and TEs [18]. Due to the recombination repression the apomixis locus is highly conserved within species, slightly divergent between species within genera and highly divergent between genera [65]. On the basis of comparative mapping using multiple grass genomes as reference, no chromosome areas were identified as syntenic to the apomixis locus that is common to the several apomictic species studied to date. In fact, the apomixis locus of *Paspalum simplex* (ACL) was syntenic, at the map level, to a conserved chromosome area that is located in telomeric position on chromosome 12, 8, 3 and 4 of rice, *Sorghum*, *Setaria* and *Brachypodium*, respectively, and near to the centromere on chromosome 1 of maize [58]. Conversely the apomixis locus in *Pennisetum squamulatum* (ASGR) showed frequently small- but not large-scale synteny with chromosomal regions of rice, *Setaria* and *Sorghum* genomes [94]; in *Tripsacum dactyloides,* RFLP markers linked to apomixis mapped to chromosome 6 of maize and in *Cenchrus ciliaris* to chromosome 6 of *Sorghum* [95] and in *Brachiaria brizantha,* to chromosome 5 of maize [96]. More recent comparative mapping studies in *Brachiaria* showed that, although high levels of synteny and collinearity were found between the ASGR of *P. squamulatum* and the apomixis locus of this genus, the apomixis carrier chromosome was identified as homologous to chromosome 1 and 5 of *Setaria* in *Brachiaria humidicola* and *B. decumbens*, respectively [97]. Furthermore, fine mapping of ASGR-carrier chromosome outside the ASGR of *P. squamulatum* showed a clear synteny with chromosome 2 of *Setaria* and *Sorghum* [98]. Taken together, these information strongly indicate that the apomixis locus has been evolved as a single event within an evolutionary lineage and was spread among plant families through hybridisation or phylogenetic diversification [99]. Nevertheless, at least two independent events have been identified within Panicoideae subfamily one in *Paspalum* and the other in *Pennisetum/Cenchrus/Brachiaria*.

Do different routes of evolution reflect functional differences in apomixis? Although apomixis in *Paspalum* and *Pennisetum* are both of the aposporous type, followed by the parthenogenic development of the egg cell, substantial differences do exist for the development of endosperm. In both cases, fertilization of the central cell is necessary for the formation of viable endosperm and seed; however, whereas in *Paspalum,* both unreduced polar nuclei are fused so as to get a C ratio of embryo:endosperm of 2:5, considering a reduced sperm to fertilize central cell, in *Pennisetum/Cenchrus* only one polar nucleus is fertilized yielding a 2:3 C ratio, as well as in sexual races of the same complex. This means that in apomictic *Paspalum* spp., the endosperm balance number of 2:1 of maternal:paternal genome ratio in the endosperm that has been proven to be essential for proper seed development, especially in grasses [100,101], might be relaxed in these species. As a matter of fact, apomictic tetraploid strains of *P. notatum* tolerated several assortments of genomes in the endosperm whereas sexual strains yielded viable seeds only when endosperm held the canonical 2m:1p ratio of parental genomes in the endosperm [102]. On the basis of findings that rare triploid individuals do occur in regions in which apomictic tetraploids are grown in sympatry with sexual diploids [103] and of the above reported studies of comparative mapping, it has been hypothesized that the ACL of *Paspalum* originated in an unstable chromosomal region of the ancestral grass genome where: (i) sex related genes were grouped by gene migration in the same genome context during speciation, (ii) a polyploidization event (through an intermediate triploid bridge) induced locally further small scale rearrangements that, in turn, (iii) caused lack of chromosome pairing and local sequence divergence and a block of recombination. In this view gene migration and polyploidization are critical steps toward emerging apomixis in this genus. In one recently proposed model to explain the successful plant gene mobility in angiosperms, Bennetzen and Wang [104] highlighted the criteria for a protein-encoding gene to conserve its function after transfer to a new location of the genome: these are its (i) small size, (ii) conservation of cis regulatory elements and (iii) landing in a no repressive genomic context (Small Insulated Genes Move Around (SIGMAR)). Furthermore, the most likely mechanism for insertional gene mobility is retroduplication by LTR retrotransposons, involving a reverse transcription of a transcript that causes elimination of introns. Among the apomixis-linked genes analysed by Galla et al. [58], *PsORC3* fits the SIGMAR condition for gene mobility well, as it is a small gene (2 Kb), it expresses a functional protein and completely lacks introns [56]. The ortholog of this gene was neither in the syntenic area of rice nor even in the same chromosome. Similarly, in *Brachypodium* it is far from the homology area of the apomixis locus though in the same chromosome. Conversely, in the *Panicoideae* subfamily (*Zea mays*, *Setaria*, *Sorghum*), the *PsORC3* ortholog was in the same syntenic group as the apomixis-linked BACs of *Paspalum*. Furthermore, since a variant allele of this gene was found in the sexual counterpart of the ACL of *P. notatum* (Ortiz et al. in preparation) this indicates that migration of this gene preceded the development of apomictic reproduction in *Paspalum*. Another intrinsic characteristic of the ACL of *Paspalum* probably peculiar of this genus is that the genes contained in this locus are transcribed in a coordinated manner as an operon-like gene cluster, so as these genes are constitutively transcribed [105] and expressed in reproductively committed cells and tissues as sense and antisense transcripts [56,58]. Although the conventional view of gene action in biology has centred on the dogma DNA→mRNA→protein, there are mounting evidences that many processes related to development are regulated by portions of the genome not necessarily linked to their coding capacity [106]. In particular, mRNAs that are transcribed from the reverse strand of a gene (natural antisense transcripts, NATs) are traditionally considered nonfunctional noise [107]. More recently, the development of the next sequence generation technology and the gaining of knowledge on the gene silencing, contribute to change this opinion revealing that an extensive number of NATs have a functional role. NATs have been surveyed in Arabidopsis [108], rice [109], wheat [110] and several legumes [111] and involved in response of abiotic stresses [112,113] and developmental transitions [114,115,116]. The mechanism of action of NATs are still not fully understood [117], although in many cases their role is in the repression of their sense cognate transcripts [118]. Our findings show that apomixis-linked genes are expressed as sense and antisense transcripts in cell lineages of sexual ovules, whereas their expression of both transcripts was strongly down regulated in the same cells of apomictic ovules. Coordinated down or up regulation of both sense and antisense transcripts derived from NAT pairs has been reported under water stress conditions in maize [112], suggesting that “sexual” and “apomictic” alleles may interact each other as NAT gene pairs (i.e., gene pairs that express transcripts that are overlapping and complementary). The nature of this interaction is generally poorly understood, although a possible link between antisense transcripts and chromatin modification has been proposed [108,119]. Again, we take *PsORC3* as a case study. This gene exists in three copies In *P. simplex* of which one, (*PsORC3a*) is specific of the ACL and is expressed constitutively as sense and antisense transcripts in nucellus and polar nuclei. *PsORC3a* might act a dominant negative regulator of the gene machinery responsible of the correct genome set up in the endosperm through antisense silencing of downstream acting genes that are common between sexual and apomictic genotypes of the *P. simplex* agamic complex. If this hypothesis will be confirmed we then have a further evidence of the Koltunow theory according to which apomixis acts dominantly over the sexual state [120] as well as the male phenotype is superimposed over female condition in some dioecious systems [121]. Finally, as Polegri et al. [105] pointed out, the genetic determinants of apomixis (i.e., *PsORC3a*) will likely be able to trigger apomixis only if the sexual recipient genome is preadapted to regulate expression of genes acting downstream (other copies of *PsORC3*) of the apomixis-linked factors. In a more general view, evolutionary, structural and functional findings serve modulate strategies aimed at introgressing apomictic reproduction in sexual crops.

## 6. Concluding Remarks

Apomixis, understood as asexual reproduction by seed (agamospermy), is thought to be repeatedly emerged in sexual lineages and eventually derived from sexual genotypes by means of mutations or modifications of genes or genomes. Despite being faced by several researchers over many years, nowadays the origin of apomixis still represents an unsolved problem, as it may be either evolved from sex [29] or the other way around [122]. Despite a number of disadvantages associated with apomixis, including narrow genetic variation and high mutation accumulation, asexually reproducing plants are highly adaptable and stable from an evolutionary perspective, mainly in terms of stabilization of polyploid genomes. Nowadays, an increasing body of evidence suggests that apomixis may be regarded as a consequence of sexual breakdown, rather than as a recipe for clonal success. As a matter of fact, ancestral sexual traits, such as meiosis and syngamy, show strong phylogenetic continuities among either closely related or distant taxa. The latter does not seem to be the case for apomixis since there are great discontinuities within orders, families within orders, genera within families, and even species within genera. Considering the two most widespread routes to originate unreduced gametophytes, i.e., apospory and diplospory, they do not appear to have a common ancestor in monocots and eudicots. In addition, it is also evident that the diplosporous condition involves a less radical departure from the normal sexual pattern that does the aposporous one. Hence devising a universal evolution model of apomixis may be unrealistic suggesting as a consequence that its different routes are likely controlled by distinct genetic factors showing distinct molecular functions. Here we have considered the basal angiosperm lineages, the ANA grade, as through the analysis of their reproduction systems and barriers is possible to re-evaluate the significance of apomixis in the evolution of angiosperm plants. As a matter of fact, within the basal ancestors and early-branching angiosperms, such as *Amborellaceae*, *Nymphaeales* and *Austrobaileyales* outcrossing systems are prevalent, some species have common adaptations to promote selfing, whereas only two species, *Trithuria incospicua* and *T. filamentosa* have been documented as putative agamospermous, even if several other modes of asexual propagation are exhibited, including vegetative proliferation by tubers, stolons and other propagules. We know that gametophytic apomixis, by means of either apospory or diplospory, is especially common in genera of the families of *Rosaceae*, *Asteraceae* and *Poaceae*. Because in these families apomixis is phylogenetically clustered above the genus level, it has been already argued that some clades, including closely related species that originated from a common ancestor within eudicots and monocts, may be preadapted and inclined to let apomixis evolve more easily. Increasing access to plant genome sequences have offered us the opportunity to compare genes related to germline initiation in sexual and apomictic species of modern genomes with reconstructed founder ancestors of flowering plants (emerged around 214 million years ago during the late Triassic era). In particular, we investigated master regulatory elements of sexual plant reproduction, including genes essential for both male and female meiosis during sex organ development (i.e., genes that control functional changes of the cell fate of sporocyte initials and the commitment to and initiation/progression of sporogenesis in both male and female organs). Since the formation of haploid spores marks the initiation of the gametophytic phase of the life cycle of all vascular plants, ANA grade genomic data assisted us to understand the evolutionary forces that have shaped this master gene in sexual plant genomes and allowed us to gain insight into how it is organized and structured in apomictic plant genomes. As we know that functional apomixis requires not only apomeiosis but also parthenogenesis and, in plants, endosperm formation, the unreduced embryo sacs should possess egg cells that are believed to be epigenetically programmed, as apomicts, for the other two features. Therefore, we also investigated the studies meant to identify the epigenetic mechanisms that may control the reproductive switch.

In conclusion, we carried out a re-evaluation of the origin of apomixis in order to deepen knowledge on such asexual mode of reproduction, which seems only supposed in one basal angiosperm family (Hydatellaceae), while it clearly occurs in different forms and variants as well as in many unrelated families of monocots and eudicots. Overall findings strengthen the hypothesis that apomixis as a whole may have evolved multiple times in angiosperm plant evolution following different developmental pathways deviating to different extents from sexuality. Recent developments on the genetic control of apomixis in model species allowed us to shed additional light on the antagonist theories of gain- and loss-of-function over sexuality.

## Supplementary Materials

The following are available online at https://www.mdpi.com/2073-4425/11/8/859/s1, Figure S1: Pairwise identity percentages between SPL protein from *Arabidopsis thaliana* (AT4G27330) and other 11 SPL-like amino acid sequences retrieved, from *Zea mays* (KY110964.1)*, Oryza sativa* (LOC_Os01g11430.1), *Hypericum perforatum* (OBUPD-D1 Hpctg51499), *Brassica oleracea* (Bol013057)*, Malus domestica* (MD11G1234600), *Prunus persica* (Prupe.4G192500.1), *Vitis vinifera* (VIT_219s0014g03940.1), *Lactuca sativa* (Lsat_1_v5_gn_0_5400), *Amborella trichopoda* (XP_006833114.1), *Nymphaea colorata* (XP031473161.1), *Nymphaea thermarum* (KAF3782288.1).
