# Supplementary material for: A Reappraisal of the Evolutionary and Developmental Pathway of Apomixis and Its Genetic Control in Angiosperms
Source: Genes (Basel). 2020 Jul 28;11(8):859. doi: 10.3390/genes11080859 (PMC7466056; doi:10.3390/genes11080859)
Supplement: Supplementary file 1 [file genes-11-00859-s001.pdf]

genes

MDPI

Supplementary Figure

	Osativa	Zmays	Atrichopoda	Nthermarum	Ncolorata	Hperforatum	Lsativa	Boleracea	Athaliana	Vvinifera	Ppersica	Mdomestica
Osativa		40.7%	11.5%	14.1%	14.4%	13.0%	12.4%	12.0%	12.7%	15.7%	13.1%	11.6%
Zmays	40.7%		12.0%	11.8%	12.3%	12.1%	12.1%	10.7%	12.8%	13.4%	14.8%	13.6%
Atrichopoda	11.5%	12.0%		25.3%	25.0%	17.3%	17.6%	17.9%	18.2%	21.7%	23.7%	20.9%
Nthermarum	14.1%	11.8%	25.3%		97.8%	18.8%	18.5%	18.2%	19.7%	22.9%	22.2%	18.4%
Ncolorata	14.4%	12.3%	25.0%	97.8%		18.8%	18.5%	17.9%	20.3%	23.2%	22.7%	18.7%
Hperforatum	13.0%	12.1%	17.3%	18.8%	18.8%		18.2%	20.9%	20.2%	21.2%	21.4%	18.0%
Lsativa	12.4%	12.1%	17.6%	18.5%	18.5%	18.2%		16.5%	17.4%	23.6%	22.8%	20.4%
Boleracea	12.0%	10.7%	17.9%	18.2%	17.9%	20.9%	16.5%		64.0%	25.6%	20.3%	17.4%
Athaliana	12.7%	12.8%	18.2%	19.7%	20.3%	20.2%	17.4%	64.0%		25.2%	19.7%	17.0%
Vvinifera	15.7%	13.4%	21.7%	22.9%	23.2%	21.2%	23.6%	25.6%	25.2%		30.9%	28.5%
Ppersica	13.1%	14.8%	23.7%	22.2%	22.7%	21.4%	22.8%	20.3%	19.7%	30.9%		56.0%
Mdomestica	11.6%	13.6%	20.9%	18.4%	18.7%	18.0%	20.4%	17.4%	17.0%	28.5%	56.0%	

Figure S1. Pairwise identity percentages between SPL protein from *Arabidopsis thaliana* (AT4G27330) and other 11 SPL-like amino acid sequences retrieved, from *Zea mays* (KY110964.1), *Oryza sativa* (LOC_Os01g11430.1), *Hypericum perforatum* (OBUPD-D1 Hpctg51499), *Brassica oleracea* (Bol013057), *Malus domestica* (MD11G1234600), *Prunus persica* (Prupe.4G192500.1), *Vitis vinifera* (VIT_219s0014g03940.1), *Lactuca sativa* (Lsat_1_v5_gn_0_5400), *Amborella trichopoda* (XP_006833114.1), *Nymphea colorata* (XP031473161.1), *Nymphea thermarum* (KAF3782288.1)
